# Diverging in vitro inflammatory responses toward *Streptococcus uberis* in mouse macrophages either preconditioned or continuously treated with β-hydroxybutyrate

**DOI:** 10.3168/jdsc.2020-0038

**Published:** 2021-03-19

**Authors:** T.H. Swartz, B.J. Bradford, L.K. Mamedova

**Affiliations:** 1 Department of Animal Science, Michigan State University, East Lansing 48824; 2 Department of Animal Sciences and Industry, Kansas State University, Manhattan 66506

## Abstract

Hyperketonemia is a common condition in early-lactation dairy cows that has been associated with an increase in the risk of infectious disease. Recent mouse studies have elucidated an anti-inflammatory effect of the ketone body β-hydroxybutyrate (BHB). Therefore, the objective of this study was to determine whether BHB altered inflammatory responses in macrophages challenged with the common mastitis pathogen *Streptococcus uberis*. A secondary objective was to determine whether the inflammatory response to the *S. uberis* challenge was dependent on whether BHB was present in the medium during the challenge (i.e., preconditioned vs. continuous treatment). Two cell culture experiments were conducted. In the first experiment, mouse macrophages (RAW 264.7 line) were preconditioned with BHB (0, 0.6, 1.2, and 1.8 m*M*) for 24 h; the medium was then replaced with a standard cell culture medium, and the cells were challenged or not with *S. uberis* for an additional 6 h. In the second experiment, a similar protocol was used; however, cells were preconditioned with BHB (0, 0.6, 1.2, and 1.8 m*M*) for 24 h, the medium was replaced with fresh medium containing the same concentration of BHB, and cells were either challenged or not with *S. uberis* for 6 h. In both experiments, relative transcript abundance of cell membrane receptors (*Tlr2* and *Gpr109a*), cytokines (*Il1b, Il10, Tnf*, and *Tgfb1*), and chemokines (*Cxcl2* and *Ccl5*) were determined using quantitative real-time PCR and normalized against the geometric mean of *Hprt* and *B2m*. Data were analyzed using a linear mixed model, and orthogonal contrasts were conducted to examine the effect of *S. uberis* challenge and BHB treatment. *Streptococcus uberis* activated the macrophages, noted by greater transcript abundance of analyzed genes. Intriguingly, in both experiments, the *S. uberis* challenge increased expression of *Gpr109a*, which encodes a receptor that is ligated by BHB. Paradoxically, preconditioning macrophages with BHB increased transcript abundance of the immunosuppressive cytokine *Tgfb1* and increased that of the neutrophil chemoattractant *Cxcl2*. Preconditioning decreased *Tlr2* and tended to decrease *Il10* transcript abundance. In opposition to the preconditioning experiment, continuous treatment of BHB during the *S. uberis* challenge linearly increased abundance of *Tlr2* and *Il10* transcripts. Continuous BHB treatment also increased expression of *Il1b*. In conclusion, BHB treatment altered macrophage inflammatory responses during an *S. uberis* challenge; however, the direction of this response was dependent on whether BHB was added to the medium during the *S. uberis* challenge. Future studies should be conducted using bovine macrophages and in vivo approaches to examine BHB effects during an *S. uberis* challenge.

## Highlights

• β-Hydroxybutyrate preconditioning reduced *Tlr2* and tended to reduce *Il10* expression.
• Continuous β-hydroxybutyrate treatment increased *Tlr2* and *Il10* expression.
• Diverging responses due to the timing of BHB treatment suggest opposing mechanisms.

## Graphical Abstract

**Figure:**
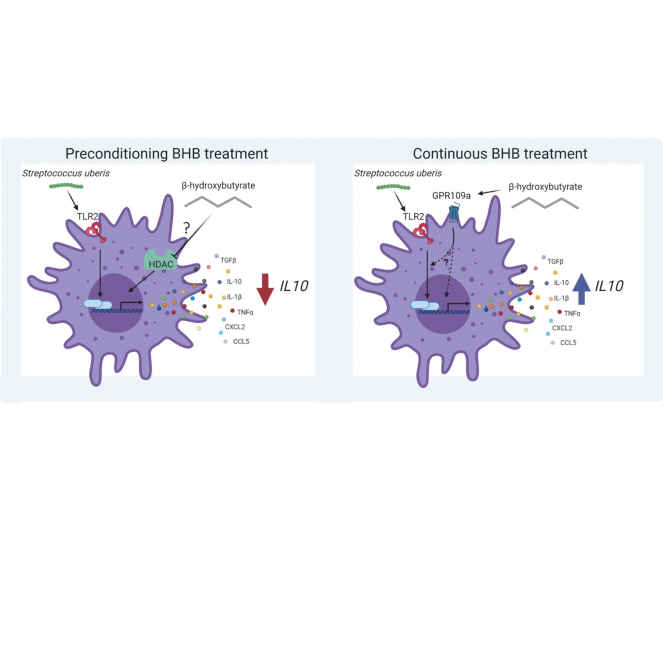
**Summary:** Elevated ketone concentrations have been associated with mastitis in postpartum dairy cows. Therefore, we sought to determine the effect of a ketone on macrophage inflammatory responses during a *Streptococcus uberis* challenge. When cells were pretreated with a ketone before *S. uberis* challenge, mRNA abundance of a membrane receptor for a bacterial cell wall component was reduced. However, when cells were treated during the challenge, the opposite was observed. These responses could help explain the association of ketosis with mastitis.

Mastitis is the most common and costly disease in the dairy industry. The incidence of clinical mastitis is dramatically greater during the first few weeks of lactation (van den Borne et al., 2010; Hammer et al., 2012). At the beginning of lactation, a depression of feed intake occurs simultaneously with an increase in energy demand, resulting in negative energy balance. Consequently, dairy cattle mobilize fat reserves, and some of these fatty acids are transported to the liver for ATP production. However, not all fatty acids are completely oxidized, resulting in the production of BHB, a major ketone body. Hyperketonemia is defined as an abnormal increase in circulating ketone bodies that may result in the health disorders more commonly known as clinical and subclinical ketosis. These disorders have been associated with greater incidence in early lactation dairy cattle of infectious diseases, including mastitis (Oltenacu and Ekesbo, 1994; Raboisson et al., 2014). Alarmingly, a large field study reported that 43% of postpartum dairy cows have subclinical ketosis (≥1.2 m*M* BHB; McArt et al., 2012), underscoring the potential implications of subclinical ketosis for infectious disease incidence.

Numerous studies have demonstrated that BHB impairs immune function (Suriyasathaporn et al., 2000). Indeed, BHB treatment reduced the ability of neutrophils to kill bacteria (Grinberg et al., 2008), impaired lymphocyte proliferation (Franklin et al., 1991) and antibody production (Nonnecke et al., 1992), and reduced phagocytosis in bovine milk macrophages (Klucinski et al., 1988a). *Streptococcus uberis* is a common environmental mastitis pathogen (Oliver, 1988; Jayarao et al., 1999; Riekerink et al., 2008). Coinciding with negative energy balance, environmental streptococci have a greater IMI rate during the first month of lactation compared with the remainder of the lactation (Todhunter et al., 1995), suggesting that hyperketonemia may be a risk factor for *S. uberis* infections. Curiously, *S. uberis* infections result in an uncontrolled inflammatory response due to sustained migration of neutrophils (Thomas et al., 1994); however, this influx of leukocytes is generally not well correlated with reductions in bacterial counts (colony-forming units; Pedersen et al., 2003; Rambeaud et al., 2003; Bannerman et al., 2004). Macrophages are critical innate immune cells responsible not only for killing bacterial pathogens but also for recruiting additional immune cells to the inflammatory site. Although debatable (Denis et al., 2006), past research has suggested that macrophages play a more pivotal role than neutrophils in *S. uberis* clearance (Thomas et al., 1994). Therefore, the objective of this experiment was to examine the effect of BHB on inflammatory mediators from macrophages during an *S. uberis* challenge. A secondary objective was to determine whether the inflammatory response to the *S. uberis* challenge was dependent on whether BHB was present in the medium during the challenge (i.e., preconditioned vs. continuous treatment). We hypothesized that BHB would attenuate inflammatory responses demonstrated by shifts in cytokine transcript abundance toward an anti-inflammatory profile.

A wild-type strain of *S. uberis* (provided by Dr. Petersson-Wolfe, Department of Dairy Science, Virginia Tech, Blacksburg) was originally isolated from a dairy cow with mastitis and stored in 10% skim milk at −80°C. Bacteria were streaked on an esculin blood agar plate and incubated overnight. Five colonies were then cultured in Todd-Hewitt broth and incubated for 7 h at 37°C on an orbital shaker (200 rpm). The bacterial suspension was pelleted by centrifugation at 1,600 × *g* for 20 min, washed with sterile PBS, and resuspended in Dulbecco's modified Eagle medium (Sigma-Aldrich) containing 1% l-glutamine and 10% heat-inactivated fetal bovine serum. Serial dilutions were used to achieve the desired concentration for challenge, and the challenge inoculum concentration (2.5 × 10^5^ cfu) was verified using drop plating onto esculin blood agar.

Mouse macrophages (RAW 264.7 line) were cultured in Dulbecco's modified Eagle medium supplemented with 1% l-glutamine, 10% heat-inactivated fetal bovine serum, and 0.2% penicillin-streptomycin. Twenty-four-well plates were seeded with 1 × 10^5^ cells per well and incubated in a humidified atmosphere for 24 h at 37°C and 5% CO_2_. To maintain a neutral pH in culture medium, BHB was added as sodium salt, and a treatment group with 1.8 m*M* added NaCl was included as an osmotic control (**OC**). Two experiments were conducted: (1) a BHB preconditioning experiment with no BHB added to the medium during the *S. uberis* challenge, and (2) a continuous BHB experiment with a 24-h preconditioned BHB treatment followed by BHB treatment during the *S. uberis* challenge. Experiment 1 assessed the effects of BHB preconditioning on macrophage inflammatory responses during an *S. uberis* challenge, whereas experiment 2 assessed the interaction of BHB treatment with *S. uberis* challenge in addition to a preconditioning effect. In experiment 1, cells were preconditioned with BHB (Sigma-Aldrich) at various concentrations (0, 0.6, 1.2, or 1.8 m*M*) for 24 h. After the 24-h incubation step, the medium was removed and fresh medium without antibiotics and with or without 2.5 × 10^5^ cfu of *S. uberis* was added for 6 h. In experiment 2, a similar protocol was used; however, cells were preconditioned with BHB for 24 h, and then the cells were challenged or not with 2.5 × 10^5^ cfu *S. uberis* by replacing the medium with fresh medium without antibiotics, but still containing the same BHB concentration as used during the preconditioning for a total of 30 h of incubation with BHB (24 h of preconditioning plus 6 h during the *S. uberis* challenge).

To measure transcript abundance, cells (n = 8 wells per treatment group) were placed on an ice pack, the medium was aspirated, and the cells were washed once with cold PBS and then lysed using a 1% 2-mercaptoethanol RLT lysis buffer (Qiagen). Cell lysates were stored at −80°C, and total RNA was isolated within 3 d of freezing using the RNeasy kit (Qiagen). RNA was quantified using spectroscopy, and purity was assessed using the 260/280 nm absorbance ratio. Complementary DNA was synthesized immediately following RNA isolation; RNA integrity was not assessed. Total RNA (0.9 and 1 µg for experiments 1 and 2, respectively) was used as a template for the reverse transcription reaction using random primers (Bio-Rad Laboratories Inc.). Quantitative real-time PCR was performed (7500 Fast Real-Time PCR System, Applied Biosystems) in duplicate with 200 n*M* gene-specific forward and reverse primers with iTaq Universal SYBR Green Supermix (Bio-Rad Laboratories Inc.). Primers were designed from mouse GenBank sequences to amplify an intron-spanning region of the gene and validated by identifying a single amplicon from the melt curve analysis. Primer efficiencies were calculated using a 5-point curve (Table 1). Transcript abundance was quantified using the relative expression ratio from Pfaffl (2001), with the geometric mean of *Hprt* and *B2m* used to normalize values.

**Table 1:** Sequence (F, forward; R, reverse), accession number, and primer efficiency for analyzed transcripts

Gene symbol	Sequence	Accession number	Mean efficiency
*B2m*	F: TAAGCATGCCAGTATGGCCG R: TGTCTCGATCCCAGTAGACG	NM_009735.3	1.30
*Ccl5*	F: TGCTGCTTTGCCTACCTCTC R: TCCTTCGAGTGACAAACACGA	NM_013653.3	1.15
*Cxcl2*	F: ACTGAACAAAGGCAAGGCTAAC R: CAGGTACGATCCAGGCTTCC	NM_009140.2	1.04
*Gpr109a* (*Hcar2*)	F: TTTGAGTCCCAGATGCACCC R: ACCCTAGGACGAAGAGCCAT	NM_030701.3	1.19
*Hprt*	F: GCAGTACAGCCCCAAAATGG R: ATCCAACAAAGTCTGGCCTGT	NM_013556.2	0.93
*Il1b*	F: TGCCACCTTTTGACAGTGATG R: TGATGTGCTGCTGCGAGATT	NM_008361.4	1.15
*Il10*	F: GGCGCTGTCATCGATTTCTC R: CTCTTCACCTGCTCCACTGC	NM_010548.2	1.22
*Tgfb1*	F: GTCACTGGAGTTGTACGGCA R: AGCCCTGTATTCCGTCTCCT	NM_011577.2	0.80
*Tlr2*	F: TGATGGTGAAGGTTGGACGG R: CCTCTGAGATTTGACCTCCTTGA	XM_006501460.3	1.19
*Tnf*	F: TAGCCCACGTCGTAGCAAAC R: ACAAGGTACAACCCATCGGC	NM_013693.3	1.02

To determine whether BHB treatment influenced cell viability, cellular metabolism was assessed using resazurin as a proxy for cell viability (Riss et al., 2004). In black-walled, clear-bottomed 96-well plates, 20 µL of a 0.15 mg/mL solution of resazurin sodium salt (Sigma-Aldrich) was added directly to RAW 264.7 cells in 200 µL of medium following BHB treatments (n ≥ 8 wells per treatment group). These plates were then incubated for another 4 h to allow conversion of resazurin to resorufin. Cellular metabolism was assessed by measuring absorbance at 570 nm using a plate reader (Synergy HTX; BioTek Instruments Inc.) and Gen5 software (BioTek Instruments Inc.). Results are expressed as a percentage of the control.

Statistical analyses were conducted using PROC GLIMMIX (SAS 9.4, SAS Institute Inc.), and each experiment was analyzed separately. The model included the fixed effects of treatment and the random effect of cell culture plate. For cell viability, orthogonal contrasts (LSMESTIMATE statement with Bonferroni adjustment) were performed to test the overall effect of BHB (OC vs. BHB treatment groups), as well as linear and quadratic contrasts to test BHB dose responses. For transcript abundance, orthogonal contrasts were performed to test the effect of *S. uberis* (unchallenged control and OC vs. OC + *S. uberis*), overall effect of BHB within *S. uberis*-challenged treatment groups (OC + *S. uberis* vs. *S. uberis*-challenged groups with either 0.6, 1.2, or 1.8 m*M* BHB treatments), as well as linear and quadratic contrasts to test BHB dose responses. To meet the assumption of normality (PROC UNIVARIATE), all response variables required natural logarithmic transformation. An outlier was defined if the observation had a studentized residual >3 in absolute value, and therefore was removed from the analysis. Significance was declared at *P* ≤ 0.05 and trends at 0.05 < *P* < 0.10.

Cellular metabolism was assessed 24 h posttreatment as a proxy for cell viability. Treatment (overall BHB effect, *P* = 0.32) did not influence cellular metabolism of resazurin (LSM ± SE expressed as a percentage of the control; CON, 100 ± 2%; OC, 95 ± 2%; 0.6 m*M* BHB, 91 ± 3%; 1.2 m*M* BHB, 87 ± 3%; 1.8 m*M* BHB, 94 ± 3%). For mRNA abundance, we first assessed the effects of *S. uberis* and BHB treatment on the geometric mean of the cycle threshold (Ct) values of *B2m* and *Hprt* to validate the internal control genes. Neither *S. uberis* nor BHB influenced the control genes (*S. uberis* effect, *P* = 1.00; overall BHB effect, *P* = 1.00). As expected, *S. uberis* activated the macrophages, as shown by greater transcript abundance of *Tlr2, Gpr109a, Ccl5, Cxcl2, Il1b, Il10, Tgfb1*, and *Tnf* (all *P* < 0.01) compared with unchallenged macrophages (Figure 1). β-Hydroxybutyrate preconditioning reduced *Tlr2* (overall BHB effect, *P* = 0.04) and tended to reduce *Il10* (overall BHB effect, *P* = 0.07) transcript abundance. Conversely, BHB preconditioning increased *Cxcl2* (overall BHB effect, *P* = 0.02) and increased *Tgfb1* in a dose-dependent manner (overall BHB effect, *P* < 0.01; linear BHB effect, *P* = 0.02).

**Figure 1:**
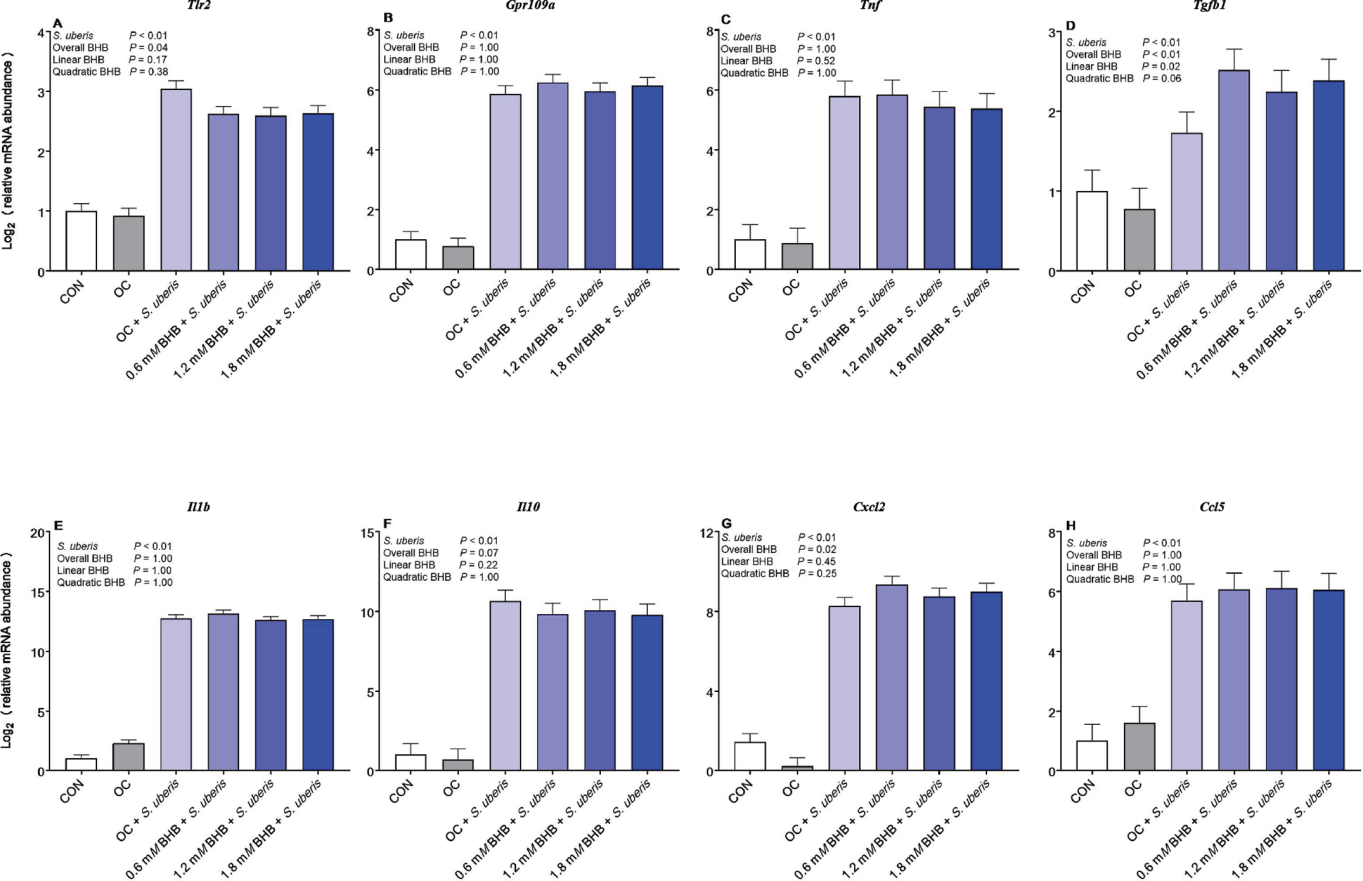
Effect of BHB preconditioning during a *Streptococcus uberis* challenge on transcript abundance of genes involved in inflammation. β-Hydroxybutyrate preconditioning reduced *Tlr2* (A) and tended to reduce *Il10* (F), while increasing *Tgfb1* (D) and *Cxcl2* (G) transcript abundance during an *S. uberis* challenge. Treatment groups (n = 8 replicates/treatment group) include control (CON), osmotic control (OC; 1.8 m*M* NaCl), 0.6 m*M* BHB, 1.2 m*M* BHB, and 1.8 m*M* BHB. Treatments were applied 24 h before challenge; the medium was then removed and replaced with fresh medium containing 2.5 × 10^5^ cfu of *S. uberis* for 6 h. β-Hydroxybutyrate was not added to the medium during the 6-h challenge.

Cellular metabolism was assessed 30 h following BHB treatment as a proxy for cell viability. Treatment (overall BHB effect, *P* = 1.00) did not influence cellular metabolism of resazurin (LSM ± SE; CON, 100 ± 3%; OC, 91 ± 5%; 0.6 m*M* BHB, 93 ± 3%; 1.2 m*M* BHB, 96 ± 3%; 1.8 m*M* BHB, 95 ± 3%). Furthermore, neither *S. uberis* nor BHB influenced the geometric mean of the Ct values of the internal control genes *B2m* and *Hprt* (*S. uberis* effect, *P* = 0.63; overall BHB effect, *P* = 1.00). As in the previous experiment, *S. uberis* activated the macrophages, noted by greater transcript abundance of *Tlr2, Gpr109a, Ccl5, Cxcl2, Il1b, Il10, Tgfb1*, and *Tnf* (all *P* < 0.01) compared with unchallenged macrophages (Figure 2). In opposition to the preconditioning experiment, continuous BHB treatment increased *Tlr2* (overall BHB effect, *P* = 0.03; linear BHB, *P* = 0.04) and *Il10* (overall BHB effect, *P* = 0.05; linear BHB, *P* = 0.02) transcript abundance in a linear manner. Similarly, BHB treatment dose-dependently increased *Il1b* abundance (overall BHB effect, *P* < 0.01; linear BHB, *P* < 0.01).

**Figure 2:**
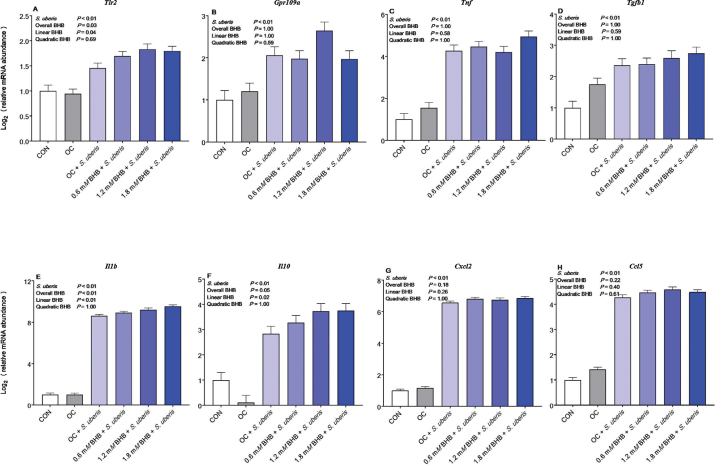
Effect of continuous BHB treatment during a *Streptococcus uberis* challenge on transcript abundance of genes involved in inflammation. β-Hydroxybutyrate treatment dose-dependently increased *Tlr2* (A), *Il1b* (E), and *Il10* (F) transcript abundance during an *S. uberis* challenge. Treatment groups include control (CON), OC (osmotic control; 1.8 m*M* NaCl), 0.6 m*M* BHB, 1.2 m*M* BHB, and 1.8 m*M* BHB. Treatments (n = 8 replicates/treatment group) were applied 24 h before challenge; the medium was then removed and replaced with fresh medium containing 2.5 × 10^5^ cfu of *S. uberis* for 6 h but still containing the same BHB concentration as used during the preconditioning (24 h of BHB preconditioning plus 6 additional hours of BHB treatment during *S. uberis* challenge).

Numerous studies have examined the effect of BHB on bovine immune function using in vitro methods (Klucinski et al., 1988a,b; Franklin et al., 1991; Nonnecke et al., 1992; Suriyasathaporn et al., 1999; Grinberg et al., 2008; Ster et al., 2012; Carretta et al., 2020). The general consensus of these studies is that BHB impairs functionality of immune cells, through reduced phagocytosis, antibody production, proliferation, and dysregulated chemotaxis, depending on the immune cell discussed. Our study is the first to show that BHB altered inflammatory responses in macrophages challenged with *S. uberis* and that these effects are dependent on the presence of the BHB in the medium during the challenge.

In our study, BHB preconditioning reduced *Tlr2* and tended to reduce *Il10* in a dose-dependent manner, although no effect was found on the proinflammatory cytokines *Tnf* and *Il1b*. Similar results were reported with butyrate preconditioning of macrophages, which enhanced antimicrobial functions toward *Salmonella*, reduced the anti-inflammatory cytokine IL-10, and had no effect on TNF-α or IL-1β protein or transcript abundance (Schulthess et al., 2019). The likely mechanism behind the BHB preconditioning effect is inhibition of histone deacetylase (Shimazu et al., 2013; Newman and Verdin, 2014). As the name suggests, histone deacetylases are enzymes that remove acetyl groups from histones (Gregoretti et al., 2004) and, by doing so, these enzymes regulate gene expression.

β-Hydroxybutyrate preconditioning increased *Tgfb1* and *Cxcl2* during an *S. uberis* challenge. Transforming growth factor-β is a pleiotropic cytokine involved in many cellular functions, including proliferation, differentiation, and regeneration, as well as suppressing inflammatory responses (Weiss and Attisano, 2013), whereas CXCL2 is a chemoattractant for neutrophils produced by macrophages in response to *S. uberis* (Günther et al., 2016). To the best of our knowledge, this is the first study to report BHB preconditioning effects on *Tgfb1* and *Cxcl2*. Considering the various alterations in cytokine transcript abundance noted in this experiment, it is difficult to reconcile the results because many of these differentially expressed cytokines have opposing effects. As such, in vivo studies are needed to better illustrate whether these BHB effects can alter mammary gland defenses during an *S. uberis* infection.

Continuous BHB treatment dose-dependently increased *Tlr2, Il1b*, and *Il10*. Toll-like receptor 2 is a critical cell membrane receptor for identification and elimination of *S. uberis* (Li et al., 2020). Although it is not clear why BHB treatment increased *Tlr2*, we speculate that this could be a response to impaired phagocytosis, as has been previously shown with ketone bodies (Klucinski et al., 1988a). Ultimately, a reduction in phagocytosis would result in greater *S. uberis* counts in the cell culture medium, leading to greater activation of TLR2, which would in turn drive greater transcription of proinflammatory genes including *Tlr2* and *Il1b*. An increase in the anti-inflammatory cytokine *Il10* is consistent with past studies examining BHB (Zarrin et al., 2014; Chen et al., 2018) or butyrate effects (Singh et al., 2014) during an inflammatory insult. *Streptococcus uberis* increased *Gpr109a*, which is a receptor for BHB that has known anti-inflammatory effects when activated (Blad et al., 2012) by augmenting IL-10 production (Al-odaini and Singh, 2019). This is intriguing, because these data could imply that *S. uberis* impairs the immune system's ability to kill this pathogen by increasing GPR109A expression on macrophages and subsequently driving an IL-10 response.

We found diverging effects on *Tlr2* and *Il10* between preconditioned and continuous BHB treatment of macrophages. When reading the literature evaluating treatment effects on cellular functions, it is easy to reach quick conclusions with little regard to when treatments were applied. Here, we provide a cautionary finding because the effects of BHB on inflammation-related transcripts were dependent on whether the BHB was present in the medium during the challenge. In light of these results, our data promote some reconsideration of preconditioning effects, especially because this is a popular treatment protocol used in cell culture studies. Moreover, although we find the preconditioning effects intriguing, it seems more likely that a continuous treatment would more closely reflect what occurs in vivo.

In conclusion, *S. uberis* is responsible for a large proportion of mastitis during the first month of lactation, suggesting that hyperketonemia may be a risk factor for this pathogen. Preconditioning macrophages with BHB resulted in a decrease in *Tlr2* and tended to decrease *Il10*, yet in continuously treated cells, BHB treatment increased the abundance of these transcripts. Altered cytokine transcript abundance could be indicative of the immune dysfunction that is typically seen in periparturient dairy cows. Future studies should be conducted using bovine macrophages to examine BHB effects on inflammation during a challenge, as the use of a mouse macrophage cell line may limit extrapolation to bovine mastitis. Finally, additional studies should be conducted to assess not only the effects of elevated concentrations of BHB, but also the various other metabolic changes that occur in the peripartum period.
